# The prevalence of hearing loss and use of hearing aids among adults in Germany: a systematic review

**DOI:** 10.1007/s00405-019-05312-z

**Published:** 2019-02-09

**Authors:** Jan Löhler, Leif Erik Walther, Fynn Hansen, Philipp Kapp, Jörg Meerpohl, Barbara Wollenberg, Rainer Schönweiler, Christine Schmucker

**Affiliations:** 1 Department of Otorhinolaryngology, Head and Neck Surgery, University Hospital of Schleswig-Holstein, Campus Lübeck, Maienbeeck 1, 24576 Bad Bramstedt, Germany; 2 German Study Centre for Otorhinolaryngology, Head and Neck Surgery (DSZ-HNO), Bonn, Germany; 3 Department of Otorhinolaryngology, Head and Neck Surgery, University Medicine Mannheim, University of Heidelberg, Mannheim, Germany; 4 Institute for Evidence in Medicine (for Cochrane Germany Foundation), Faculty of Medicine, University Teaching Hospital, Albert Ludwig University of Freiburg, Freiburg, Germany; 5 Department of Otorhinolaryngology, Phoniatrics and Paediatric Audiology, University Hospital of Schleswig-Holstein, Campus Lübeck, Lübeck, Germany

**Keywords:** Hearing loss, Hearing impairment, Adults, Hearing aids, Prevalence, Systematic review, Germany, incidence, CI, Cochrane

## Abstract

**Background:**

Worldwide approximately 360 million people suffer from hearing impairment, 328 million of whom are adults. Up to now there has been no systematic evaluation of any representative epidemiological data on the prevalence of hearing loss among adults in Germany. The present paper is intended to investigate this within the framework of a systematic review.

**Methods:**

A systematic literature search was carried out in electronic databases as well as by means of hand-searching. Studies published after 1975 and indicating the prevalence or incidence of hearing impairment among German adults were included. Study selection, data extraction and additional quality assessments were made by two independent reviewers.

**Results:**

By means of a systematic literature search it was possible to identify 6 sources, which provided solely cross-sectional data, whereby the reported data are based on a study population of between some hundred and 10 million people living in Germany. The prevalences ascertained showed a broad range of between 16% and 25% and varied according to age, study setting, definition of hearing loss and method of data capture. At present there are no utilizable data on the extent of the use of hearing aids.

**Discussion:**

The present review demonstrates clearly that evidence-based information relating to Germany can only be made on the basis of a clear definition of hearing loss within the framework of an up-to-date and representative epidemiological study carried out with appropriate methodology. In view of the high prevalence of illnesses causing hearing impairment and of the risks to health associated with untreated hearing impairment as well as of socio-economic costs, such an epidemiological study is of great social significance.

**Electronic supplementary material:**

The online version of this article (10.1007/s00405-019-05312-z) contains supplementary material, which is available to authorized users.

## Introduction

According to the Global Burden Disease Study of the World Health Organization (WHO), hearing impairments are one of the most common disorders in patients reducing the quality of life in most industrialized countries [1]. Worldwide approximately 5.0% of the entire population or one-third of all adults over the age of 65 would require treatment due to hearing loss [2]. New-born hearing screening programs and different registers for hearing loss, e.g., the German register for hearing disorders in children, [3] support the documentation of the prevalence, incidence, and severity of different hearing disorders in children. In adults, however, a thorough documentation, e.g., in form of a hearing screening program or another obligatory examination is lacking, even in highly developed nations. This lack of documentation—particularly in low-risk people (i.e., those without increased noise exposure)—excludes the early detection and treatment of patients affected by a gradually progressive hearing loss.

Besides vascular disorders, chronic inflammation, noise exposure, genetic susceptibility, and tumors (such as vestibular schwannoma), the physiological aging of the inner ear is often the cause of hearing impairments at ages over the fifth or sixth decade of life. This aging process is referred to as presbycusis [4]. It develops gradually and is not noticed by those affected until a much later stage when they experience difficulties in communication or even worse when they are actually excluded from communications. Particularly, untreated hearing impairments in the second half of life have a significant impact on everyday life and may increase the risk of memory loss [5], the tendency to falls [6], the risk of depressions [7] and the acceleration of dementia [8]. Presbycusis also results in social isolation and severe psychological problems including psychoses [9, 10].

In Germany, the annual costs of health disorders caused by hearing impairments are estimated at € 2.65 billion [11]. WHO estimates that unaddressed hearing loss poses an annual global cost of US$ 750 billion (http://www.who.int/news-room/fact-sheets/detail/deafness-and-hearing-loss). Although disorders associated with hearing losses are regarded as a significant medical problem, undertreatment is common, especially in the case of presbycusis due to its slow progression. For example, it was reported that only 16% of those requiring treatment are receiving an adequate intervention in form of hearing aids [12]. Taking into account that people with hearing loss can greatly benefit from the use of hearing devices (such as hearing aids), implantable hearing systems (such as active middle ear or cochlear implants) and speech therapy [13, 14], early recognition of this symptom in mandatory.

To provide an overview of the prevalence of hearing disorders in adults in an industrialized country (Germany), we performed a systematic review as part of an evidence project initiated by the German Study Centre of Otolaryngology, Head and Neck Surgery established by the German Society of Otolaryngology, Head, and Neck Surgery and the Professional Association of German Otolaryngologists [15, 16]. The aims of this review are as follows: (i) to identify all relevant studies and data sources providing prevalence and/or incidence data on hearing loss in the general population living in Germany, and (ii) to summarize these data considering their quality (risk of bias and generalizability). This approach will allow us to assess whether there is a lack of evidence-based knowledge in this otorhinolaryngologic epidemiologic research area.

## Methods

For this systematic review, we followed the reporting guidelines provided by the PRISMA statement [17]. A review protocol can be accessed from the corresponding author.

### Population and setting

Studies or other data sources providing estimates on the prevalence or incidence of hearing disorders in adults (over 18 years of age) living in Germany were included in this systematic review. Studies considering populations with specific diseases were as excluded as well as studies published prior to 1975.

### Outcomes

The following patient-relevant outcomes were addressed: (i) prevalence of hearing impairment (frequency of the disorder, i.e., proportion of afflicted adults); (ii) incidence of hearing impairment (number of new cases in a defined observation period); (iii) proportion of patients with hearing aids and/or cochlear implants.

### Study types

No restrictions were set up regarding the design of the included studies. Duplicate publications without relevant additional information were not taken into consideration.

### Literature search

A systematic literature search for studies published in the German or the English language was carried out in May 2017 in the following electronic databases: Medline, Medline Daily Update, Medline In Process, and other Non-Indexed Citations (Ovid), Web of Science (Thomson Reuters), Cochrane Library (http://www.cochranelibrary.com), ScienceDirect (Elsevier), and LIVIVO.

To identify additional studies, the ‘tables of contents’ of specialist journals not listed in electronic databases were screened [e.g., TW Kopf Hals (Head and Neck), Zeitschrift für Allgemeinmedizin (German Journal of Family Medicine), Gesundheitswesen (Healthcare), Zeitschrift für Audiologie (Journal of Audiology)]. We also searched the bibliographies of relevant studies to identify further citations manually. A search was also made for ongoing or completed but not yet published studies in the registers for clinical trials (clinicaltrials.gov) and the German study register (Deutsches Register für klinische Studien, DRKS, http://www.drks.de). Moreover, the websites of the Robert-Koch-Institute (RKI, http://www.rki.de), the National Association of Statutory Health Insurance Physicians (Kassenärztliche Bundesvereinigung, KBV, http://www.kbv.de), the National Association of Statutory Health Insurance Funds (http://www.gkv-spitzenverband.de) and the German Federal Statistics Office (http://www.destatis.de) were searched for further prevalence or incidence data.

The search strategy used in Medline (Ovid) is presented in the Online Appendix. Search strategies for other databases were modified to meet the requirements of each database.

### Study selection

Two reviewers (JL, FH or CS) screened the titles and abstracts of all reports identified by electronic searches and hand searching. Afterward, we obtained full-text copies of all potentially relevant articles and again, two reviewers (JL, FH or CS) assessed them for inclusion.

### Data extraction

The above-mentioned authors also independently carried out data extraction and performed the methodological assessment of the included studies. Any discrepancies in the data extraction were resolved by discussion. The following data were extracted from the studies: (i) details of publication characteristics, i.e., bibliographical data (e.g., author(s), year of publication), information on the design of the study, exact geographical area of data collection, period of data collection, age and number of adults included, and the definition of the measured hearing impairment (including the assessment method); (ii) *outcome data*, i.e., details of prevalence and / or incidence of the measured hearing impairment as well as information on the proportion of people with hearing impairments treated with hearing aids.

### Assessment of risk of bias and generalizability of results

The risk of bias (internal validity) and generalizability (external validity) was assessed according to pre-defined criteria which were developed by our group considering published epidemiological literature [18] and internal discussions. The assessment of risk of bias was based on: (i) the reliability of data capture, i.e., whether the prevalence of hearing impairments were judged by the respondents themselves (e.g., in an interview or by means of a questionnaire; high risk of bias), or whether the studies applied standardized audiometric procedures (e.g., pure-tone audiogram; low risk of bias); (ii) definition/specification of the measured hearing loss, i.e., whether the hearing impairment was defined after standardized criteria (e.g., in accordance with WHO criteria; low risk of bias) or whether no adequate definition was used (e.g., this refers primarily to a self-reported hearing impairment; high risk of bias); (iii) the completeness of data, i.e., whether the whole study sample (all recruited people) was considered when data were analyzed (low risk of bias) or whether data were missing (e.g., due to drop-outs; high risk of bias). Generalizability assessment was based on the selected study sample, i.e., whether the study sample was preselected with regard to certain characteristics. For example, when only patients of one ORL practice were investigated or when only people from one region or city in Germany were considered, generalizability was judged as low. In case the study included a broad-ranging sample, generalizability was judged as high. If no judgment could be made owing to missing information, the quality assessment was classified as “unclear”.

For data extraction and the assessment of the risk of bias and generalizability of results, we relied on information provided in the individual studies.

## Results

### Systematic literature search

**Fig. 1:**
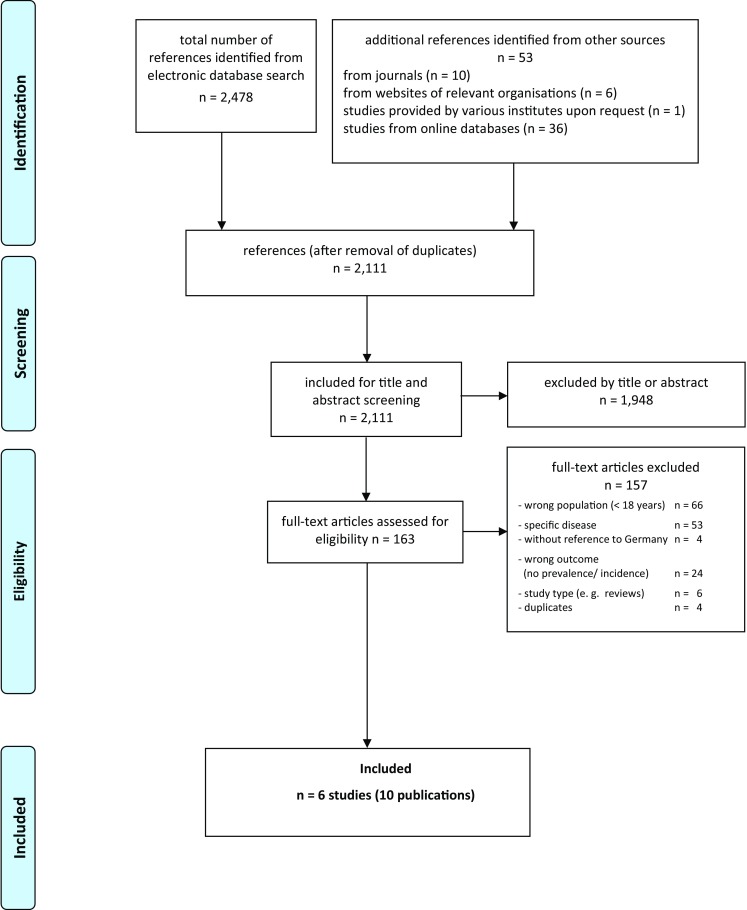
Prisma flowchart of the literature research [17]

The systematic literature search identified 2478 references. Additionally, we identified 53 references by hand searching, including 36 registry entries referring to ongoing or completed and not yet published studies [clinicalstrials.gov (*n* = 16) and the DRKS register (*n* = 18)]. After deducting the duplicates, there remained 2111 references. These references were assessed on the basis of their title and abstract. In total, 1948 references were excluded because they did not address our research question. Finally, 163 potentially relevant references were included for full-text screening. From these, six studies (10 publications) provided data on the prevalence of hearing disorders. The study flow is presented in Fig. 1 (PRISMA flowchart [17]).

### Study characteristics

Table 1 outlines the study characteristics. Of note, one study was identified in the DRKS register and was not published at the stage when the current review was conducted (DRKS-ID: DRKS00009783 [19]). Furthermore, the study of von Gablenz et al. [20] is based on two publications, known as HÖRSTAT [21] and Aalen report [22]. Moreover, the data of HÖRSTAT were used for two further publications [23, 24].

**Table 1:** Study characteristics

References	Study design	Time of data capture	Geographical region	Study population			Definition of hearing impairments	Test method
				Patient population	Ages (years)	Number		
HörMAT [19]	Cross-sectional	2016–2017	Berlin and Bochum (based on patient data)	Consecutive patients consulting clinics	50–75	943	Hearing loss: inability to hear one of the frequencies 0.5, 1, 2, 4 kHz at ≥ 25 dB	PTA up to 8 kHz + subjective self-assessment MAT [27]
von Gablenz et al. [20]	Cross-sectional	HÖRSTAT: 2010–2012 Aalen report: 2008–2009	Aalen, Oldenburg, and Emden	Randomized samples	18–97	3150 (HÖRSTAT: 1866/Aalen report: 1239)	Hearing loss: defined according to WHO^a^ extrapolation of data to provide incidence rates for the years 2015/20/25	PTA-4^b^: 0.5, 1.0, 2.0, 4.0 kHz + subjective self-assessment
RKI [26]	Cross-sectional	2012–2013	Germany	Randomized sample from residents’ registration office	≥ 18	Approx. 26,000	Subjective self-assessment of hearing (telephone survey: no, slight, major difficulties in hearing)	A standardized questionnaire (telephone survey)
Neubauer and Gmeiner [11]	Cross-sectional (retrospective)	2008	Germany (based on patient data)	Randomized sample of patient data	18–90 +	10,600,000	Hearing loss: defined according to ICD	Routine data (KBV data): invoicing data of patients attending GPs or ENT doctors
Sohn and Jörgenhaus [12]	Cross-sectional	2001	North Rhine-Westphalia (based on patient data)	Randomized sample of patients of 11 randomly selected GPs	> 14	2031	Hearing loss: inability to hear one of the frequencies 0.5, 1, 2, 3, 4 kHz at 40 dB (SD 10^c^), if pathological than followed by SD 21^d^	Audiometry SD 10^c^ + SD 21^d^, subjective self-assessment (interview)
Stange [25]	Cross-sectional	1984–1985	Former West Germany	Randomized sample of households, selection of persons by Kish selection grid^e^	15–75	2778 (2699)^f^	Hearing loss: inability to hear one medium/low frequency at 30 dB; inability to hear one high frequency at 38 dB Air conduction and bone conduction: 0.25, 0.5, 0.75, 1, 1.5, 2, 3, 4, 6, 8 kHz	Audiometry SD 21^d^ + subjective self-assessment (interview)

*ENT* ear, nose and throat, *GP* general practitioner, *HL* hearing loss, *ICD* International Statistical Classification of Diseases and related health problems, *KBV* Kassenärztliche Bundesvereinigung (National Association of Statutory Health Insurance Physicians), *PTA* pure-tone audiometry, *SD* steps of decibel

^a^Normal hearing: PTA-4 ≤ 25 dB HL, slight impairment: 25 dB < PTA-4 ≤ 40 dB, moderate impairment: 40 dB < PTA-4 ≤ 60 dB, severe impairment: 60 dB < PTA-4 ≤ 80 dB, profound impairment: PTA-4 > 80 dB

^b^Pure-tone audiometry: averaging the air-conduction tone threshold of the better hearing ear at the frequencies 0.5, 1.0, 2.0 and 4.0 kHz

^c^10 frequencies (0.5–4 kHz) at 20 and 40 dB (10 steps overall), for screening measurement at 40 dB only

^d^Eight frequencies (0.5–8 kHz) in the range of − 10 to 90 dB in 5 dB steps (21 steps overall)

^e^Procedure according to which each of the persons living in the household has the same probability of being selected

^f^2778 test persons were included in the publication, however, prevalence data base on 2699 people. The study provides no information on the remaining 79 test persons

### Characteristics of the study population

The study identified in the DRKS register (HörMAT) limited the age of the included population and included adults between 50 and 75 years of age [19]. The remaining studies included a wide age range. Beginning at 14 [12], 15 [25] and 18 [11, 20, 26] to 75 and 90 years of age or even higher (Table 1). Sample sizes ranged from just below 1000 [19], up to over several [12, 20, 25] and multiple tens of thousands [26]. Outstanding, one study provided prevalence data on the basis of more than 10 million people [11].

### Characteristics of the geographical region

Three studies gathered their data throughout Germany [11, 25, 26], whereby one of them was restricted to former West Germany and West Berlin [25]. The others provided data limited to individual regions in Germany (North Rhine-Westphalia) [12], the towns Aalen, Oldenburg, and Emden in northwestern Germany [20] or to the cities Berlin and Bochum [19]. Additionally, von Gablenz et al. [20] extrapolated data on hearing loss for the entire population of Germany up to the year 2025.

### Measurement and definition of hearing loss

The RKI reported prevalence data based on a telephone survey (i.e., a subjective self-assessment of more than 25,000 thousand people) [26]. The remaining studies provided data on hearing loss based on objective measurements (audiometry). However, besides an audiometric test, some studies also performed a subjective self-assessments by questionnaires [12, 20, 25] or used the recently developed Mini-Audio-Test (MAT [19, 27]). In detail, von Gablenz et al. [20–24] (the study which is associated with four publications) provided prevalence data based on pure-tone audiograms (PTA). Hearing loss was averaged over the four main frequencies 0.5, 1.0, 2.0 and 4.0 kHz (PTA-4) and limited to the better hearing ear. Thereby, WHO criteria were applied: normal hearing defined as PTA-4 ≤ 25 dB hearing level (HL); slight hearing impairment defined as 25 dB < PTA-4 ≤ 40 dB HL; moderate hearing impairment defined as 40 dB < PTA-4 ≤ 60 dB HL; severe hearing impairment defined as 60 dB < PTA-4 ≤ 80 dB HL; and profound hearing impairment (bordering on deafness) defined as PTA-4 > 80 dB HL [28]. In addition, speech-audiometry was carried out using the Götting Sentence Test (Göttinger Satztest, GÖSA) and the Triple-Digit Test; however, those results were not provided in the corresponding publications [21, 23, 24]. HörMAT also used PTA measurements and defined hearing loss as the inability to hear one frequency between 0.5 and 4.0 kHz at ≥ 25 dB [19]. Sohn and Jörgenhaus [12] defined hearing loss as the inability to hear one frequency between 0.5 and 4.0 kHz at ≥ 40 dB. In the study of Stange [25], the individual hearing impairment was defined as the inability to hear one frequency between 0.25 and 8.0 kHz at 30 dB and/or 38 dB. In contrast, Neubauer and Gmeiner [11] utilized the international statistical classification of diseases and related health problems (ICD) coding transmitted for invoicing purposes to the KBV (so-called routine data) to derive prevalence data.

### Prevalence of hearing impairment

Data on the prevalence of hearing loss are shown in Table 2. Based on a telephone survey, the RKI reported in 2012 that 21.5% of the German adult population suffer from hearing impairment [26]. Approximately 19.0% of people reported a minor (following a conversation with minor difficulties) and 2.7% a major impairment (following a conversation with major difficulties) by hearing loss. Von Gablenz et al. [20–22] reported a prevalence of hearing impairment of 16.2% (> 25 dB), 11.9% (> 30 dB) and 8.4% (> 35 dB) defined after WHO criteria for adults over 18 years of age. Stratifying data after age, a hearing loss of more than 25 dB was diagnosed in 6.6% (adults between 50 and 59 years of age), 20.3% (between 60 and 69 years of age), 42.3% (between 70 and 79 years of age) and 71.5% (adults over 80 years of age) [20, 21]. Prevalence data based on a subjective self-assessment for the same age groups were 25.1%, 31.5%, 44.1%, and 56.9%, respectively. This study also calculated an estimate for new cases with hearing impairment per year for the entire German population. This estimate lies between 150,000 and 160,000 new cases per year up to the year 2025. In HörMAT the prevalence of hearing loss at 25 dB or more was 46.6% (in hospitalized patients without known ear disorders between 50 and 59 years of age) and 77.8% (in patients over 60 years of age). The prevalence of hearing loss based on a subjective self-assessment was 51.1% (patients between 50 and 59 years of age) and 41.0% (among those over 60 years of age) [19]. Sohn and Jörgenhaus [12] diagnosed a hearing loss of 40 dB or more in 19% of patients consulting a general practitioner (GP) for different reasons. When the same population completed a questionnaire to assess their ‘subjective’ perceived hearing loss, the prevalence rate decreased to 14%. Of note, the study sample includes patients from the age of 14 years without providing age-dependent data. Stange [25] reported a prevalence of hearing loss of 26.8% (objectively measured at 30 dB or more in the left ear) and 20.6% (based on a subjective self-assessment) among people living in former West Germany over 15 years of age. Using ICD criteria, Neubauer and Gmeiner [11] estimated prevalence of hearing impairments of approximately 10% in a wide range of patients consulting GPs or ENT specialists for different indications (over 70 years of age) and up to 15% (among those over 80 years of age).

**Table 2:** Prevalence data

References	Age (years)	Subjective self-assessment		Audiometry	
		Methods	Prevalence (*n*) (%)	Methods	Prevalence (*n*)
HörMAT [19]	50–59	MAT: at least 2 points	51.1	PTA hearing loss: inability to hear one of the frequencies 0.5, 1, 2, 4 kHz at < 25 dB	≥ 25 dB: 46.6%
	60–75	MAT: at least 3 points	41.1		≥ 25 dB: 77.8%
von Gablenz et al. [20]	18 to ≥ 80	Questionnaire	26.4	PTA-4 (better ear), defined according to WHO	> 25 dB: 16.2% > 30 dB: 11.9% > 35 dB: 8.4%
				Extrapolation of data for the years 2015/20/25	The increase of 150,000–160,000 hearing-impaired persons per year
	18–29		7.7	PTA-4 (better ear), defined according to WHO	–
	30–39		13.6		–
	40–49		20.9		–
	50–59		25.1		> 25 dB: 6.6%
	60–69		31.5		> 25 dB: 20.3%
	70–79		44.1		> 25 dB: 42.3%
	≥ 80		56.9		> 25 dB: 71.5%
RKI [26]	> 18	Telephone survey: minor difficulties	18.8	–	
		Telephone survey: major difficulties	2.7		
Neubauer and Gmeiner [11]	19–49	–		Hearing loss: defined according to ICD-10	3.0%
	50–54				5.2%
	55–59				6.6%
	60–64				8.1%
	65–69				9.6%
	70–74				11.7%
	75–79				13.6%
	80–84				14.9%
	85–89				15.0%
	≥ 90				13.3%
	19 to ≥ 90			Extrapolation (self-calculated for the entire adult population living in Germany 2008, 61.9 million)	6.6%
Sohn and Jörgenhaus [12]	> 14	Questionnaire	14.0	Audiometry SD 21, inability to hear one of the frequencies 0.5, 1, 2, 3, 4 kHz	≥ 40 dB: 19%
Stange [25]	15–75	Questionnaire/interview	20.6	Audiometry SD 21, inability to hear one medium/low frequency at 30 dB; inability to hear one high frequency at 38 dB; air conduction and bone conduction: 0.25, 0.5, 0.75, 1, 1.5, 2, 3, 4, 6, 8 kHz	≥ 30 dB: 26.8%

SD 21 see Table 1

*ENT* ear, nose and throat, *GP* general practitioner, *HL* hearing loss, *ICD* International Statistical Classification of Diseases and related health problems, *KBV* Kassenärztliche Bundesvereinigung (National Association of Statutory Health Insurance Physicians), *PTA* pure tone audiometry

### Prevalence of people with hearing aids

**Fig. 2:**
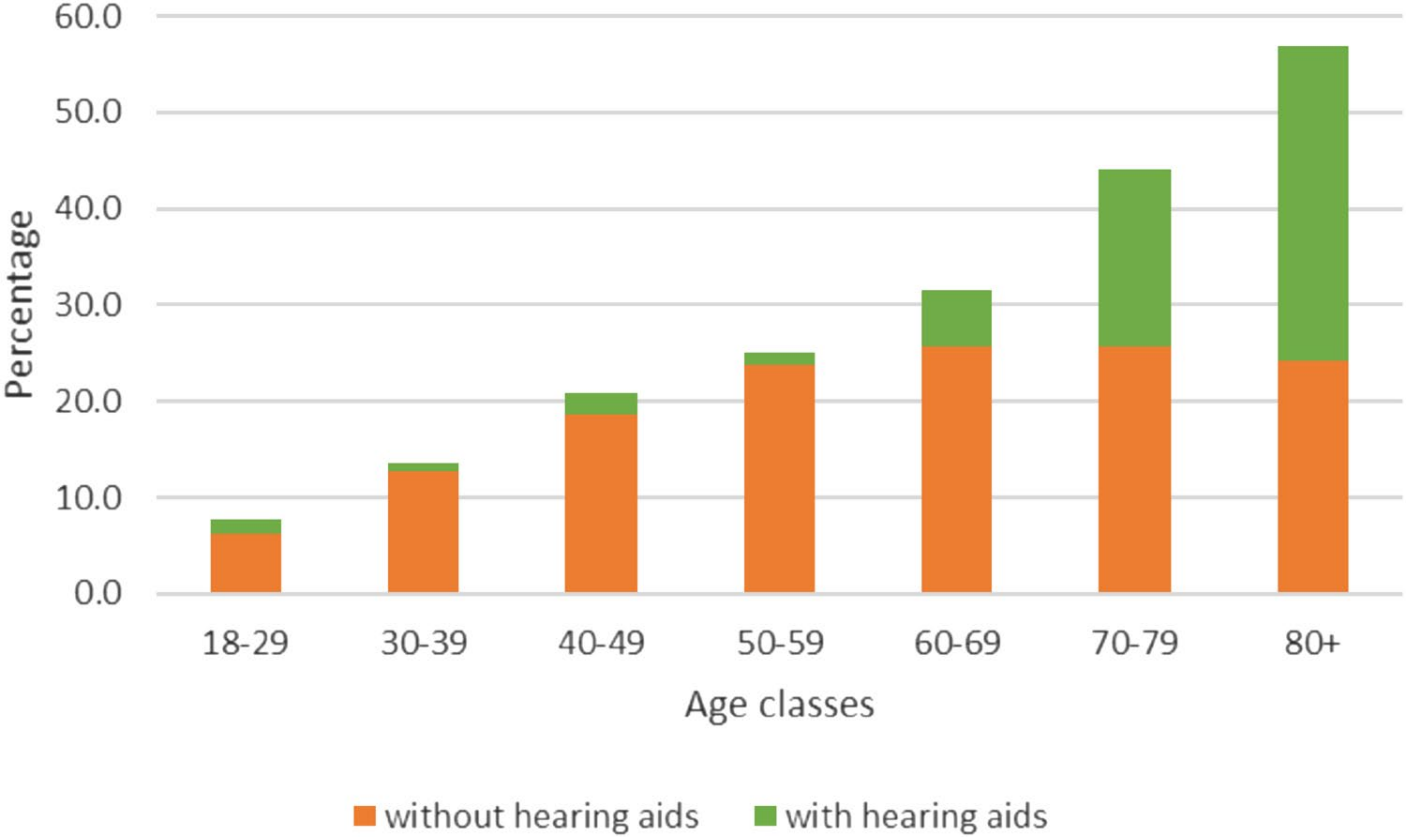
Age class-dependent percentage of hearing loss and use of hearing aids [20]

Only two studies provided data on the prevalence of hearing aids in Germany [12, 20]. HÖRSTAT [21] a publication related to the study of von Gablenz et al. [20] reported that 1.4% (age 18–29 years), 0.8% (age 30–39 years), 2.3% (age 40–49 years), 1.2% (age 50–59 years), 5.8% (age 60–69 years), 18.3% (age 70–79 years), and 32.6% (age 80 + years) of patients with hearing impairments use a unilateral or bilateral hearing aid (Fig. 2 [20]). Overall, 6.5% of the included study population used hearing aids. Sohn and Jörgenhaus [12] stated that all cases in their study suffering from moderate to severe hearing impairment were wearing hearing aids. We did not identify any study reporting data on the prevalence of implantable hearing devices or cochlear implants in the general population.

### Methodological quality

**Fig. 3:**
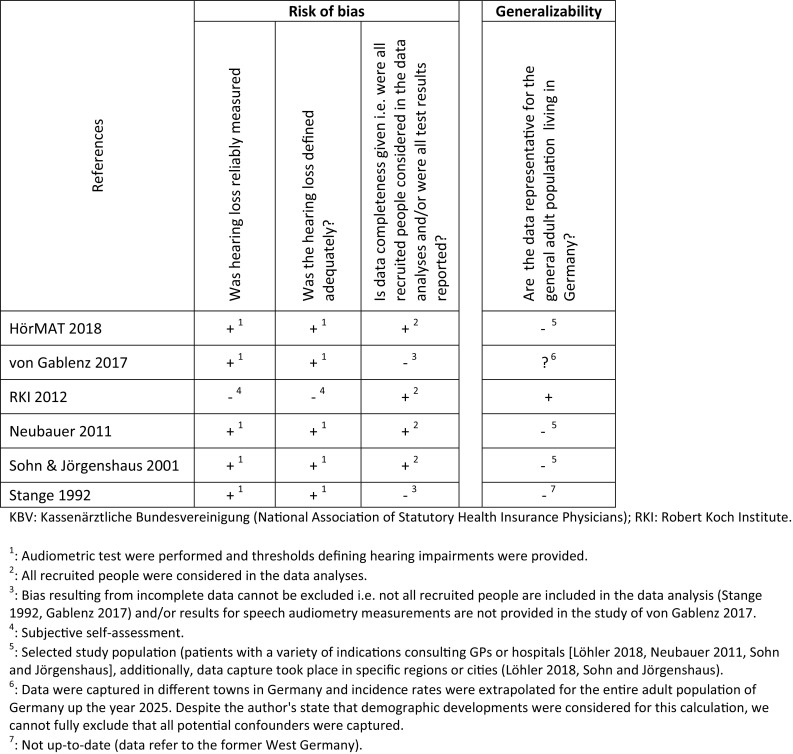
Assessment of included studies

The methodological assessment is shown in Fig. 3.

### Risk of bias

The nationwide health survey carried out by the German RKI is susceptible to a high risk of bias as no objectively measurable threshold values can be taken into account with this subjective self-assessment by telephone [26]. The remaining primary studies used standardized audiometric procedures (e.g., tone audiogram) and defined hearing loss either after WHO or other standardized criteria [11, 12, 19, 20, 25]. With the exception of two studies [20, 25], data completeness was given.

### Generalizability

Data for the entire population of Germany were provided by three studies [11, 25, 26]. However, one of these studies [25] was conducted in 1985, therefore, the reported data refer to former West Germany and are not up-to-date anymore. The other two studies are either based on prevalence data collected by a telephone survey excluding valid data (high risk of bias, see above) [26] or reflect diagnoses from patients consulting GPs or ENT specialists for different indications including hearing impairment [11]. The remaining studies collected data in specific regions, towns or cities in Germany [12, 19, 20]; two of these also included a study sample that consulted GPs or hospitals for different medical indications limiting the generalizability to the entire “general” population [12, 19]. Of note, von Gablenz et al. [20] also reports incidence extrapolations for the entire adult population of Germany up the year 2025. Despite the author’s state that demographic developments were considered for this calculation, we cannot fully exclude that all potential confounders were captured.

## Discussion

### Principal findings

Only a limited number of studies are available providing prevalence estimates of hearing impairment for the general population in Germany. Moreover, the current epidemiological data are either associated with risk of bias or lacking generalizability. Overall, prevalence data on hearing impairment for the German population showed a wide variety ranging between 16 and 25% depending on the age, the study setting, the definition of hearing impairment and the method how hearing impairment was measured. For example, the reported hearing impairment was either based on (i) frequency-specific measurements by PTAs [20], (ii) on a self-assessment (by telephone interviews) [26] or (iii) retrospectively recorded using routine data (ICD coding) [11].

Five primary studies claim that their data are representative for the entire population living in Germany [11, 12, 20, 25, 26], but a critical methodological assessment did not (fully) prove these study conclusions. For example, one of these studies was conducted in West Germany in the years 1984 and 1985 [25]. Since that time there have been major changes with regard to the national territory, the demographic composition of the inhabitants and the federal directives for the provision of medical aids. Thus, current representativeness of these study data is lacking. Another recent study reporting the age-dependent prevalence rate is limited to specific regions in Germany. Although this study extrapolated data to a national and European cohort to provide incidence rates up to the year 2025, methodological flaws and uncertainties resulting from any data extrapolation limit their validity [20]. The study from the RKI used randomized samples from residents’ registration offices throughout Germany. However, the reported prevalence estimates of 18.8% (for minor hearing impairments) and 2.7% (for major hearing impairments) are based on telephone surveys which are associated with flawed data captures [26]. Furthermore, people with known severe hearing impairment were excluded by the study author resulting in an underestimated estimate for major hearing difficulties. Another study carried out in general practitioners’ offices by Sohn and Jörgenshaus in the year 2001 utilized so-called quota sampling to achieve representativeness for Germany. Despite these approaches to control for prognostic factors, the study population consists of patients with a wide range of pre-existing conditions including ear disorders consulting a physician. Any transferability to all German inhabitants is therefore very questionable [12]. Moreover, this study includes an unknown number of adolescents aged over 14 years of age. Considering that hearing disorders are less pronounced in younger than in older people, the reported overall prevalence rate may be underestimated. Furthermore, the study using ICD codes from a randomized sample of patient data to reflect diagnoses made by physicians reported an averaged prevalence rate (over all adult ages) of 6.6% [11]. However, this estimate may be over- or underestimated because of two reasons: (i) beside the symptom hearing loss, ICD coding also includes the actual cause for this symptom and (ii) only patient data from those seeking medical advice because of subjectively perceived complaints were considered. Taking into account that patients suffering from hearing loss are often not aware of their impairment (or even deny it) and, therefore, do not consult a physician, the given prevalence rate may be underestimated [29].

An estimate of the magnitude of these unrecorded cases may be provided by studies comparing prevalence data obtained by subjective self-assessments and making use of PTAs [12, 19, 20, 25]. For example, HörMAT showed that approximately half of those over 60 years of age suffering from hearing loss were not aware of this impairment (41% (subjective self-assessment) versus 78% (PTA, ≥ 25 dB)) [19]. In the study of von Gablenz et al. [20] people over 80 years of age underestimated their hearing impairment (57% (subjective self-assessment) versus 72% (PTA, ≥ 25 dB)). The same observations were made in two other studies including much younger people (form the age of 14 or 15 up to 75 years of age or even more): 14% (subjective self-assessment) versus 19% (PTA, ≥ 40 dB) [12] and 20.6% (subjective self-assessment) versus 26.8% (PTA, ≥ 30 dB) [25]). Lacking information regarding the methodology of the subjective self-assessment excludes any quantification in terms of the severity of the perceived hearing loss.

We identified only two studies reporting on treatment coverage. However, none of these studies allowed us to judge the proportion of patients not wearing hearing aids although it would be indicated [12, 20]. There is no literature available on treatment coverage by implantable hearing devices and cochlear implants in the general population. Furthermore, indications for pertinent operations are still a matter of current scientific discussion [14].

### Ongoing studies

In the ongoing German National Cohort (GNC) men and women aged between 20 and 69 are clinically examined in 18 study centers throughout Germany [30]. In total 40,000 participants will also be investigated by speech audiometry [30, 31]. This study will provide further data on the prevalence of hearing loss in Germany. Owing to speech audiometry and its known limitations the full extent of hearing impairment in Germany will most likely not be captured.

### Strengths and limitations of the systematic review

Overall, an epidemiological systematic review provides a useful approach to capture prevalence data on a certain clinical condition. However, some challenges in relation to the methodological assessment of the primary studies with respect to the risk of bias and the generalizability of the results exist. For example, there are no well-established tools to estimate the methodological quality of such studies. Therefore, based on published epidemiological studies we developed criteria to assess both risks of bias and generalizability for the included studies which may be of high value in future epidemiological research [18]. Furthermore, the findings of our systematic review are based on a thorough and comprehensive literature search including hand searching for epidemiological studies on hearing loss in Germany. However, we are aware that our findings have several limitations related to the nature of our research work (systematic review): First, we could not identify sufficient study data to estimate the extent of hearing impairment for the general population living in Germany. Second, the risk of bias assessment revealed that the current prevalence data may be hampered due to inappropriate methods to capture such data (e.g., subjective self-assessments) and/or poor reporting making a thorough bias evaluation challenging. Generalizability of the results was limited due to hospital- or physician-based study settings based on patient data, regional or town specific participant selections and/or lacking up-to-datedness. Though the latest available prevalence data were captured between 2016 and 2017, they were limited to a special region in Germany and based on patient data [19]. In another study, valid data were collected between the years 1984 and 1985, however, these data are the only representative for former West Germany [25]. There would be even older prevalence data available, for example, data collected by Kessler and Hoffmann [32], but we decided not to include such studies because of the very long time lapse between an associated demographical, socio-economical and technical changes since then.

### Implications for clinical practice

This work has implications for researchers and those who use epidemiological data to help inform clinical and policy decisions. Overall, we revealed that (i) prevalence data on hearing loss are scarce and show methodological flaws. (ii) Prevalence data were broadly scattered reflecting different definitions of hearing loss, different methods of data capture, different not comparable age-groups and different settings; therefore, a comparison between studies was challenging. Further studies should be done by on the basis of a clear definition of hearing loss. This definition ought to be worked out in a first step. (iii) Moreover, available data are either not representative for the general in Germany living adult population (for example, most studies based their study sample on patient data or lacking currentness) or show methodological flaws limiting their validity. (iv) The rate of patients with hearing loss wearing hearing aids is very low (Fig. 2). Although this rate is increasing by age the total number of unaided patients is increasing as well (and more) with respect to the total number of hearing-impaired patients within elderly age classes. (v) We suggest a representative epidemiological study considering age-dependent frequency-specific definitions of hearing loss. This approach will also allow us to estimate the extent of the coverage of hearing aids. Particularly, in view of the high prevalence of the underlying diseases of hearing loss and the risks associated with untreated hearing impairment as well as the socio-economic costs such an epidemiological study is of great social importance.

## Electronic supplementary material

Below is the link to the electronic supplementary material.

Supplementary material 1 (DOCX 22 KB)
