# Supplementary material for: The prevalence of hearing loss and use of hearing aids among adults in Germany: a systematic review
Source: Eur Arch Otorhinolaryngol. 2019 Feb 9;276(4):945–56. doi: 10.1007/s00405-019-05312-z (PMC6426811; doi:10.1007/s00405-019-05312-z)
Supplement: Supplementary file 1 — Supplementary material 1 (DOCX 22 KB) [file 405_2019_5312_MOESM1_ESM.docx]

**Appendix 1:** **Search strategy**

**Database MEDLINE**

*Date of search: 28/04/2017*

| # | Search | Results |
| --- | --- | --- |
| 1 | "Correction of Hearing Impairment"/ | 1874 |
| 2 | exp Hearing Aids/ | 15574 |
| 3 | Cochlear Implantation/ | 5190 |
| 4 | cochlea* implant*.ti,ab,kf. | 12071 |
| 5 | ((hearing adj3 (aid* or device* or pro?thes*)) or (ear mold* or earmold*)).ti,ab,kf. | 9175 |
| 6 | or/1-5 | 23629 |
| 7 | exp Epidemiologic Studies/ | 2067010 |
| 8 | exp morbidity/ | 466294 |
| 9 | mass screening/ | 93084 |
| 10 | neonatal screening/ | 8636 |
| 11 | (prevalen* or inciden* or screen* or cross-section* or cross?ection*).ti,ab,kf. or (pra?valen* or inziden* or screen* or querschnitt*).ot. | 2007015 |
| 12 | or/7-11 | 3659689 |
| 13 | 6 and 12 | 5616 |
| 14 | exp Germany/ | 143275 |
| 15 | exp Europe/ | 1273039 |
| 16 | (german* or BRD or deutsch*).ti,ab,kf,ot. or europ*.ti,ot. | 188606 |
| 17 | (Baden-Wu?rttemberg or Bayern or Berlin or Brandenburg or Bremen or Hamburg or Hesse? or Mecklenburg or Niedersachsen or Nordrhein-Westfalen or Rheinland-Pfalz or Saarland or Sachsen or Schleswig-Holstein or Thu?ringen or Thuringia or Saxony or Rhineland or Northrhine-Westphalia or Bavaria*).ti,ab,kf,ot. | 18823 |
| 18 | (Aachen or Bochum or Bonn or Dresden or Du?sseldorf or Erlangen or Essen or Frankfurt or Freiburg or Gies?en or Go?ttingen or Greifswald or Halle or Hannover or Heidelberg or Jena or Kiel or Ko?ln or Cologne or Leipzig or Lu?beck or Magdeburg or Mainz or Mannheim or Marburg or Mu?nchen or Munich or Mu?nster or Oldenburg or Regensburg or Rostock or Homburg or Tu?bingen or Ulm or Wu?rzburg or Witten or Stuttgart).ti,ab,kf,ot. | 41429 |
| 19 | ger.la. | 840362 |
| 20 | or/14-19 | 2103253 |
| 21 | 13 and 20 | 686 |
| 22 | exp Hearing Disorders/ | 80145 |
| 23 | **Persons With Hearing Impairments/** | **2048** |
| 24 | ((hearing adj3 (disorder* or damag* or disab* or los* or impair*)) or deaf? or deafness).ti,ab,kf. | 72924 |
| 25 | or/22-24 | 103302 |
| 26 | 12 and 25 | 25400 |
| 27 | 26 and (14 or 16 or 17 or 18 or 19) | 1130 |
| 28 | "Correction of Hearing Impairment"/sn [Statistics & Numerical Data] | 22 |
| 29 | exp Hearing Aids/sn [Statistics & Numerical Data] | 191 |
| 30 | Cochlear Implantation/sn [Statistics & Numerical Data] | 108 |
| 31 | Persons With Hearing Impairments/sn [Statistics & Numerical Data] | 121 |
| 32 | exp Hearing Disorders/ep, sn [Epidemiology, Statistics & Numerical Data] | 5618 |
| 33 | or/28-32 | 5899 |
| 34 | 33 and (14 or 16 or 17 or 18 or 19) | 295 |
| 35 | exp Germany/ep [Epidemiology] | 19394 |
| 36 | 35 and (6 or 25) | 137 |
| 37 | 21 or 27 or 34 or 36 | 1663 |
| 38 | limit 37 to (english or german) | 1600 |
| 39 | exp animals/ not humans/ | 4389693 |
| 40 | 38 not 39 | 1596 |
| 41 | (animal* or rat or rats or mouse or mice or pig or pigs or piglet* or rabbit* or porc* or monkey* or macaque*).ti. | 1647564 |
| 42 | 40 not 41 | 1595 |
| 43 | remove duplicates from 42 | 1580 |
| ^a^MEDLINE 1946 to April Week 3 2017, MEDLINE Daily Update April 27, 2017, MEDLINE In Process & Other Non-Indexed Citations April 27, 2017, MEDLINE Epub Ahead of Print April 27, 2017 | | |
